# Infantile Myofibroma Presenting as a Large Ulcerative Nodule in a Newborn

**DOI:** 10.1155/2019/3476508

**Published:** 2019-09-17

**Authors:** Farooq Shahzad, Ava G. Chappell, Chad A. Purnell, Monica Aldulescu, Sarah Chamlin

**Affiliations:** ^1^Ann & Robert H. Lurie Children's Hospital, Chicago, Illinois, USA; ^2^Northwestern University Feinberg School of Medicine, Chicago, Illinois, USA

## Abstract

The differential diagnosis of a congenital cutaneous vascular-appearing mass in a newborn is broad and includes both benign and malignant tumors. We report the case of a newborn who presented with an erythematous exophytic skin nodule on the right upper leg. Excision was performed due to ulceration, concern for bleeding, and for diagnosis. Pathology revealed the mass to be an infantile myofibroma. This case highlights the importance of considering a broad differential diagnosis in a newborn with a cutaneous mass. While history, physical exam, and imaging can help diagnose some cases, a biopsy or excision is often needed to distinguish benign lesions from more concerning lesions.

## 1. Introduction

The diagnosis of vascular-appearing cutaneous masses in an infant can be challenging. We present a neonate with a vascular-appearing ulcerated skin lesion that was presumed to be a hemangioma by the referring primary care provider, and pathology later revealed it to be an infantile myofibroma (IM). A brief review of the differential diagnoses considered for this case is provided, along with the suggested management of infantile myofibroma.

## 2. Case Presentation

**Figure 1:**
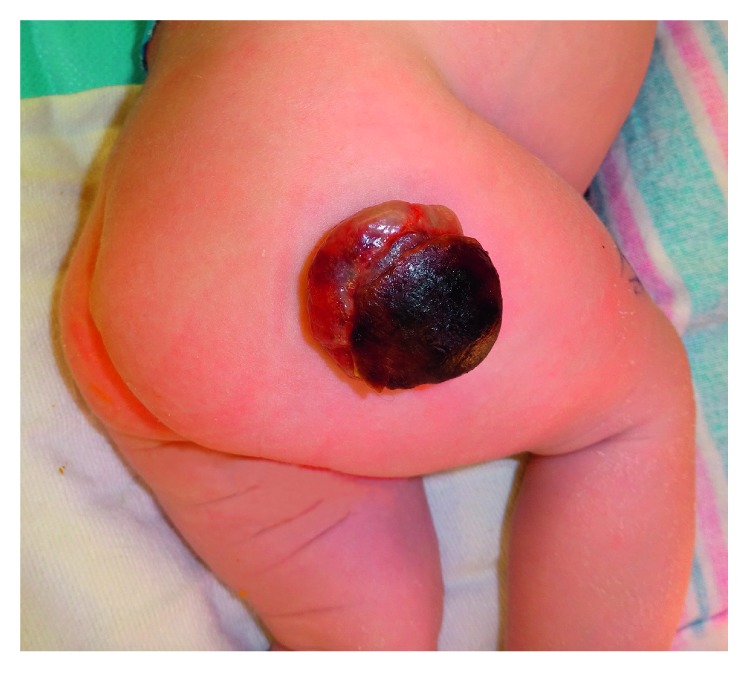
Cutaneous mass of the right thigh.

**Figure 2:**
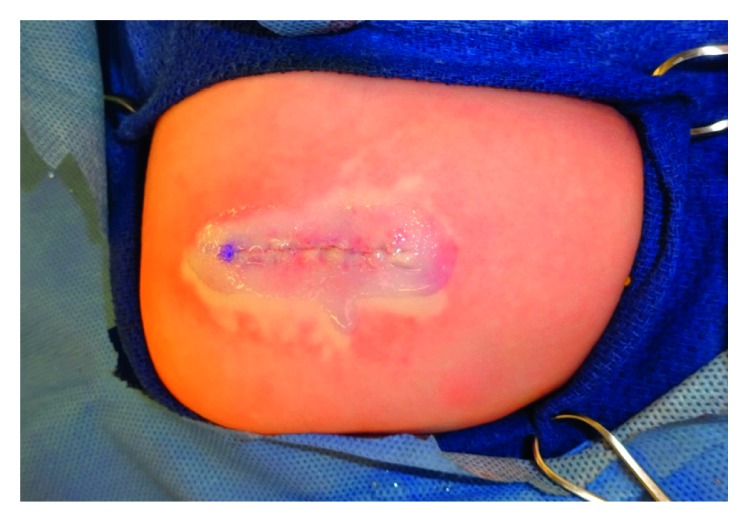
Excision of mass and primary closure.

**Figure 3:**
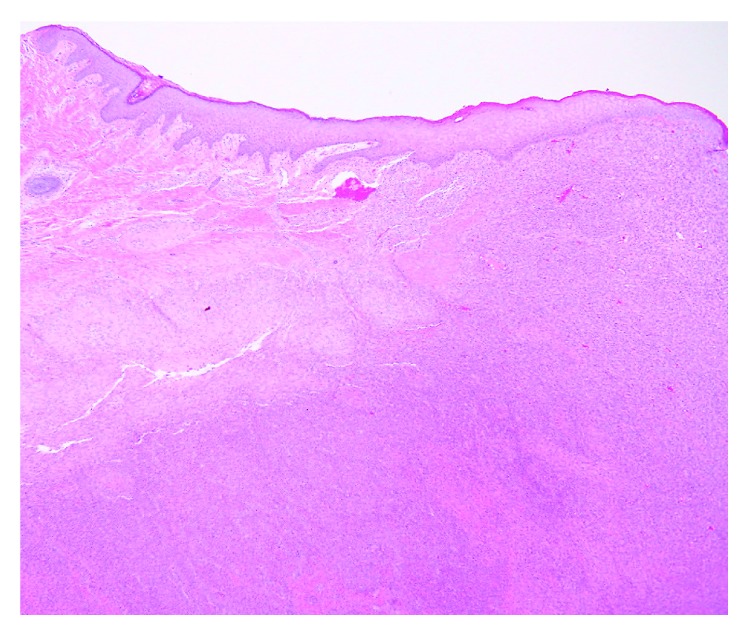
Histopathology with hematoxylin and eosin staining. Scanning view (1x magnification) shows a dermal proliferation of spindled cells with lighter and darker areas.

**Figure 4:**
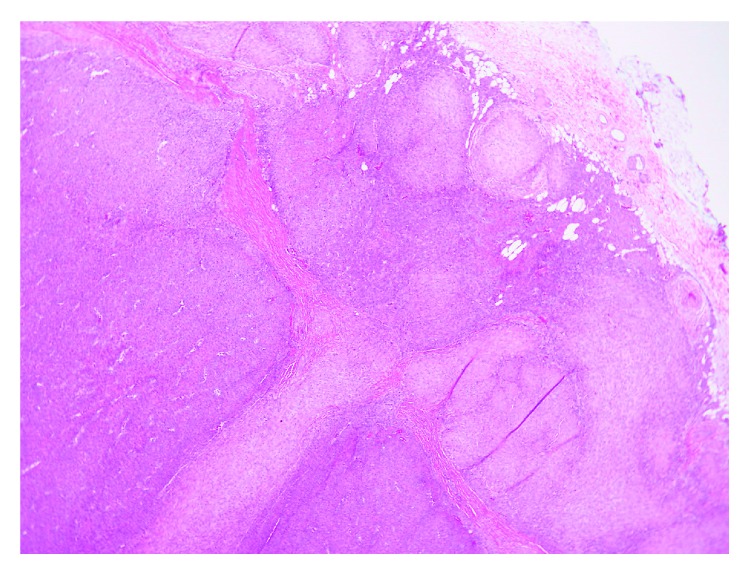
Low power view (5x magnification) shows a nodular/multinodular tumor with a zonal appearance of hypercellular areas in the center and hypocellular areas at the periphery.

**Figure 5:**
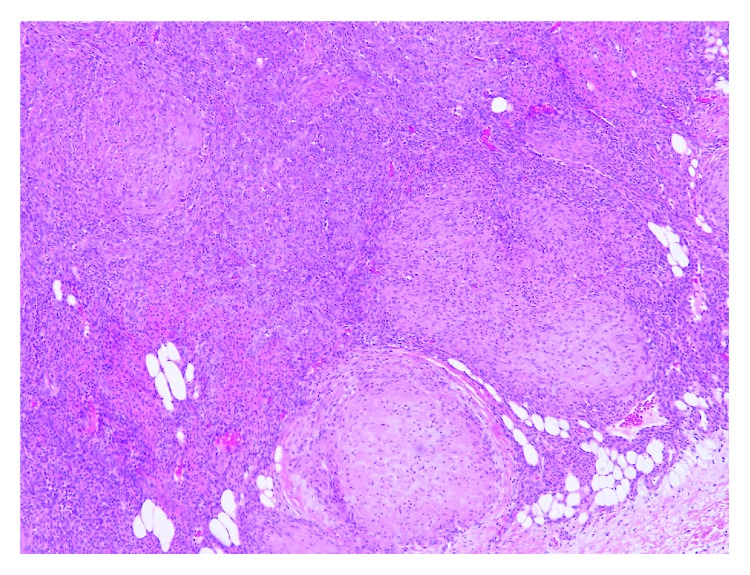
Higher power view (10x magnification) shows a multinodular, biphasic tumor with alternating hyper and hypocellular areas.

**Figure 6:**
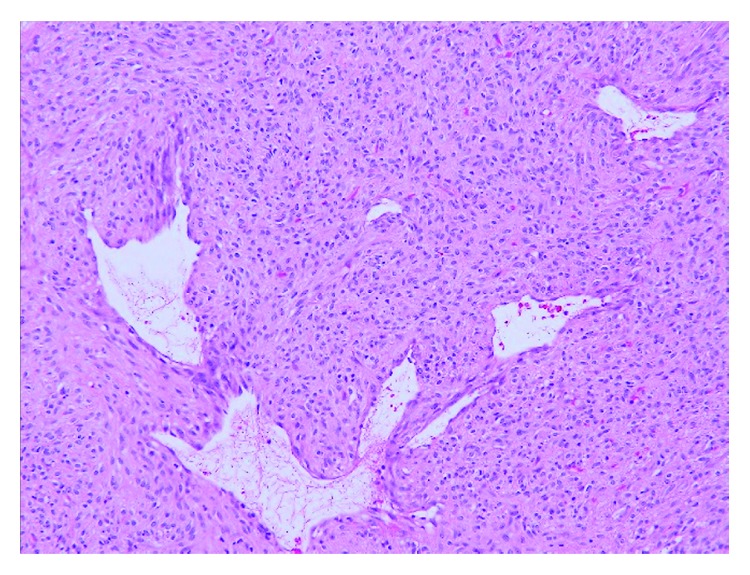
High-power view (20x magnification) shows numerous hemangiopericytoid slit-like vessels in the center of the tumor.

**Figure 7:**
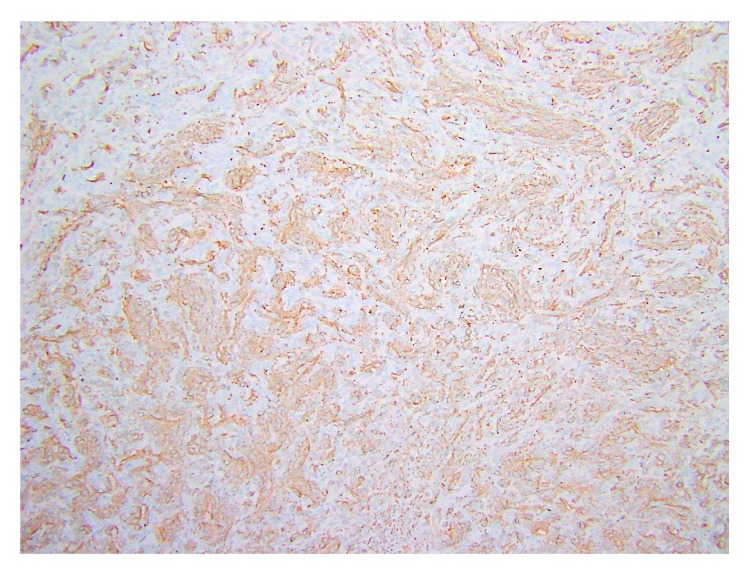
Staining for smooth muscle actin shows that myofibroblastic areas (myoid component) are positive while the fibroblastic areas (nonmyoid areas) are negative (10x magnification).

An 11-day-old male was referred for evaluation of a cutaneous mass of the right upper lateral thigh (Figure 1). The child had an uncomplicated full-term birth. The parents reported that the lesion looked like a “red ball” at birth, but over several days the surface became darker in color. The mass was nontender. His parents also noticed some blood on the diaper near the mass. On exam, the child had an exophytic erythematous nodule with overlying eschar and friable surface measuring 2 × 2 cm on the right upper lateral thigh. The appearance was not typical of a congenital hemangioma. Due to concerns about bleeding, the possibility that this might develop into a difficult-to-manage open wound, and the need for a diagnosis, the entire lesion was excised at 14 days of life. Primary closure was performed after undermining with recruitment of local tissue (Figure 2). The final pathology revealed the diagnosis of infantile myofibroma (Figures 3–7). The child's postoperative course was uneventful with no tumor recurrence at 6-month follow-up.

## 3. Discussion

Clinical diagnosis of vascular-appearing congenital skin nodules can be difficult, and often a tissue diagnosis is required. The differential diagnosis in this child included: congenital hemangioma, juvenile xanthogranuloma, pilomatrixoma, myofibroma, and fibrosarcoma.

Congenital hemangiomas are fully formed at birth, and then either undergo rapid involution (rapidly involuting congenital hemangioma or RICH), fail to involute (noninvoluting congenital hemangioma or NICH), or undergo initial rapid involution that then stops at some point (partially involuting congenital hemangioma or PICH). They often have a rim of pallor and coarse overlying blood vessels and may ulcerate, as a rare complication. Juvenile xanthogranulomas are yellow, red, or purple colored nodules that are present at birth or appear in the first year of life. They are usually solitary but can be multiple and undergo growth and ulceration. Their natural history is spontaneous regression [1]. Pilomatrixomas are common but frequently misdiagnosed [2]. They are benign tumors of the hair matrix cells that grow slowly and calcify. They appear as raised subcutaneous nodules that are skin colored, red, or blue. Usually arising in childhood, they can occasionally appear in infancy [3]. Treatment is surgical excision. Congenital fibrosarcomas are firm round skin lesions present at birth. They are slow growing, red to purple in color, fixed to deep structures and may have superficial telangiectasias or ulcerate. Biopsy provides a definitive diagnosis [4].

Of note, although neuroblastoma and nasal glioma were not included in the differential diagnosis in this case, they can present as a cutaneous, vascular-appearing masses in neonates. Nasal gliomas are frequently misdiagnosed as hemangiomas, especially by nonpediatric providers. They are collections of heterotopic neuroglial tissue that present as raised red masses on the nasal dorsum. Any midline nasal mass should raise the suspicion for a glioma or encephalocele, and CT or MRI is frequently obtained for further evaluation of the lesion and possible intracranial extension. Neuroblastoma is the most common neonatal malignant tumor, with 2/3 having metastases [5]. Cutaneous metastasis can be initial presentation of this disease with blue or purple nodules. Biopsy provides the diagnosis, which prompts a metastatic workup.

Infantile myofibromas, although rare, are the most common fibrous tumors of infancy [6]. They can arise in any part of the body, but are most commonly found in the skin and subcutaneous tissue. The majority are present at birth or arise within the first 2 years of life with a male to female ratio of 2 : 1 [7, 8]. Occasionally, they are present in adulthood [9]. Infantile myofibroma may be solitary (70 to 80%) or multicentric (20 to 30%) [8, 10]. The most common location of a solitary IM is the head and neck, followed by the trunk and extremities [8, 10]. They present as nontender, rubbery, subcutaneous, or dermal nodules of 0.5 to 7 cm in diameter that are dusky-red to purple in color [10]. Surface telangiectasias may be noted, and ulceration occurs rarely. Their appearance frequently leads to confusion in distinguishing them from congenital hemangiomas [10]. The multicentric form IM can have from a few to up to 100 lesions [11], and occasionally a large lesion is surrounded by multiple smaller lesions [8]. Important for physicians to keep in mind, approximately one third of multicentric myofibromas have visceral involvement [12].

Most cases of IM are thought to be sporadic. Familial forms of IM have been reported with autosomal dominant and recessive inheritance patterns [13, 14]. Mutations in the PDGFRB (platelet-derived growth factor receptor beta) and NOTCH3 gene have been identified in the autosomal dominant forms of the disease [15, 16]. Genetic counseling should be considered in familial cases, as future offspring may be affected.

Histopathology can provide a definitive diagnosis. IM have a characteristic histological pattern with an outer zone of spindle-shaped myofibroblasts arranged in fascicles and an inner zone of round cells with enlarged hyperchromatic nuclei surrounding thin walled hemangiopericytoma-like blood vessels. Necrosis, calcification, and vascular extension may be present in the central area. Immunohistochemical stains provide definitive diagnosis with the smooth muscle stains actin and vimentin being positive and S100 (positive in neurofibroma) and GLUT-1 (positive in infantile hemangioma) being negative.

The natural history of IM is of gradual regression, possibly due to apoptosis, over the first few years of life, although some lesions exhibit an initial phase of rapid growth [17]. Of note, bony involvement can result in pathological fractures [11]. Visceral IM portends a poor prognosis with a 33 to 75% mortality rate, primarily due to mass effect on the organs [7, 12]. Tumors have been reported to involve the pulmonary, cardiac, gastrointestinal, and central nervous systems, although they can affect virtually any organ [18–22]. The prognosis is worst with pulmonary involvement. Evaluation of visceral involvement in multicentric IM can be performed with imaging such as a skeletal survey, chest X-ray, echocardiogram, ultrasound, and CT scans [22], and whole body MRI can be performed in infants which gives an excellent evaluation of the tumors and avoids radiation [23]. Management of visceral IM is surgical excision for solitary symptomatic lesions, and recurrence rate after excision is 7 to 10% [8, 12]. Multiple lesions, unresectable lesions, and recurrences can be treated with chemotherapy ± radiation [24]. The chemotherapeutic agents that have been used include alkylating agents (cyclophosphamide and ifosfamide), vinca alkaloids (vincristine, vinblastine, and vinorelbine), doxorubicin, actinomycin-D, and methotrexate [25]. Targeted inhibitors like sunitinib [25] and crizotinib [26] are showing promise as a treatment strategy for aggressive cases.

## 4. Conclusion

This case highlights a common clinical scenario faced by pediatricians caring for newborns: accurately diagnosing a congenital vascular skin nodule. When the diagnosis of a vascular-appearing pediatric mass is not clear, further workup includes imaging studies such as ultrasound and MRI and is done by an experienced radiologist who may differentiate a solid tumor with vascularity from a congenital hemangioma. An incisional or excisional biopsy is often performed with specific immunohistochemical stains for precise diagnosis. If there is concern for systemic disease, appropriate workup should be performed. Prompt referral to an experienced pediatric dermatologist or pediatric plastic surgeon is crucial for the best plan of care.

## Abbreviations

IM: Infantile myofibroma.
